# Differences in Central and Peripheral Choroidal Thickness among the Subtypes of Age-Related Macular Degeneration in an Asian Population

**DOI:** 10.3390/jcm12165364

**Published:** 2023-08-18

**Authors:** Yosuke Fukuda, Shoji Notomi, Satomi Shiose, Kumiko Kano, Sawako Hashimoto, Kohta Fujiwara, Masato Akiyama, Keijiro Ishikawa, Toshio Hisatomi, Koh-Hei Sonoda

**Affiliations:** 1 Department of Ophthalmology, Graduate School of Medical Sciences, Kyushu University, 3-1-1 Maidashi, Higashi-Ku, Fukuoka 812-8582, Fukuoka, Japan; 2 Department of Ophthalmology, Fukuoka University Chikushi Hospital, 1-1-1 Zokumyouin, Chikushino 818-8502, Fukuoka, Japan

**Keywords:** age-related macular degeneration, pachychoroid spectrum disease, polypoidal choroidal vasculopathy, pachychoroid neovasculopathy, ultra-wide-field swept-source optical coherent tomography

## Abstract

Age-related macular degeneration (AMD) causes visual impairment in individuals who are >50 years of age. However, no study has investigated AMD when using ultra-wide-field swept-source optical coherence tomography (UWF SS-OCT). We aimed to evaluate central and peripheral choroidal thicknesses using UWF SS-OCT, and to compare these across the AMD subtypes. We included 75 eyes of patients with typical AMD (tAMD), 56 with polypoidal choroidal vasculopathy (PCV), 29 with pachychoroid neovasculopathy (PNV), and 12 with retinal angiomatous proliferation (RAP). To compare choroidal thicknesses in the central and peripheral choroids, we established subfields of <3 mm, <9 mm, and 9–18 mm from the fovea. PNV patients were significantly younger than those with tAMD (*p* = 0.01). The choroidal thicknesses of PNV were significantly greater than that of tAMD in all subfields (*p* < 0.01), and choroidal thickness significantly correlated with age and axial length in all subfields (*p* < 0.05). Even after adjusting for age and axial length, the choroidal thickness in PNV was significantly greater than that in tAMD (*p* < 0.05). In addition, the ratio of the posterior <9 mm to a peripheral 9–18 mm choroidal thickness in PNV was significantly greater than that in tAMD (*p* < 0.01). A thickened choroid in PNV was more pronounced in the posterior choroid than in the periphery.

## 1. Introduction

Age-related macular degeneration (AMD) is the leading cause of visual impairment affecting individuals over 50 years of age worldwide, and it is increasing with the global aging trend [1]. AMD is categorized into two major types, wet neovascular AMD (nAMD) and dry atrophic AMD. Cases of nAMD is more frequently found in Asian than in Western populations [2], and several clinical manifestations of nAMD differ between Asians and Caucasians. Asian eyes often develop exudative nAMD, even in the absence of extracellular deposits, which is the hallmark of early/intermediate AMD in Western populations, namely drusen [3]. Cases of AMD lacking drusen may be associated with a pathology that is associated with a thick choroid, namely pachychoroid [4,5].

Pachychoroid is associated with several alterations involving the choroidal vasculature, such as choroidal thickening, dilated choroidal vessels (pachyvessels), underlying choriocapillaris thinning, and choroidal vascular hyperpermeability [5,6,7,8]. These features, more frequently found in Asians than in Western populations [9], are associated with the abnormality of the retinal pigment epithelium (RPE) and subretinal fluid over the thick choroid. Central serous chorioretinopathy (CSC) is well characterized among the conditions associated with choroidal vessel dilatation and choroidal thickening. Veins in the deep choroidal layer (Haller’s layer) of eyes with CSC are more dilated and are accompanied by greater subfoveal choroidal thickness than in normal eyes [10,11,12], suggesting that structural changes in choroidal vessels and their hyperpermeability might contribute to the appearance of subretinal fluid, such as pigment epithelial detachment (PED) and serous retinal detachment. In addition to CSC, pachychoroid neovasculopathy (PNV) and polypoidal choroidal vasculopathy (PCV) belong to the same disease entity, the so-called pachychoroid spectrum disease (PSD). PCV, also known as aneurysmal type 1 neovascularization, is currently considered a variant of type 1 (sub-RPE) neovascularization, which is associated with vascular dilation and the feeding vascular network between the basal lamina of the RPE and the inner collagenous layer of the Bruch’s membrane [13,14]. Type 1 lesions, which are branching vascular networks, may evolve into more active forms of neovascular proliferation and produce additional exudation [15]. PNV, which was first described by Pang and Freund, represents macular neovascularization (MNV) over the thick choroid and dilated choroidal vessels (pachyvessels). It is typically found in the absence of macular drusen, and when there is no evidence of myopic or other causes of degeneration [5,8].

Recently, a new hypothesis was proposed to explain the thickened choroid underlying CSC and AMD. Dysregulated choroidal blood outflow from the vortex vein ampulla, termed venous overload choroidopathy, has been suggested as a potential mechanism underlying PSD, which is termed venous overload choroidopathy [12,16,17,18]. In this context, analyzing wide-field choroidal thickness, including the periphery, is crucial because the dysregulation of choroidal circulation, which normally outflows through the vortex vein ampulla, may be involved in the formation of pachyvessels crossing the macula. Recent advancements in OCT technology, particularly the use of swept-source OCT (SS-OCT), allow for a more detailed and high-resolution analysis of the ultra-wide-field choroidal structure [19,20]. Indeed, central and peripheral choroidal thickness in CSC has been shown to be greater than that in the fellow eye or than that in eyes of healthy subjects when using ultra-wide-field (UWF) SS-OCT [21,22]. Moreover, scleral thickness that is measured by anterior segment OCTs in CSC eyes is greater than that in normal control eyes, indicating that thick sclera may play a role in the pathogenesis of CSC, thus contributing to the congestion of choroidal blood outflow through the vortex vein ampulla [23].

One might expect that similar choroidal pathology could be implicated in AMD, particularly PNV or PCV, which is often associated with a “thickened choroid” in Asians [24]. However, it is difficult to establish a threshold of choroidal thickness to diagnose PNV or PCV because of the significant variation in healthy individuals, where the mean subfoveal choroidal thickness is 255 μm with a relatively wide standard deviation exceeding 100 μm [25]. Moreover, factors such as age, sex, and axial length can affect choroidal thickness [26]. Indeed, subfoveal choroidal thickness may decrease by 2 μm for each year of age. Additionally, a correlation exists between axial length and subfoveal choroidal thickness, particularly in individuals aged >50 years [27]. Therefore, when interpreting choroidal thickness data, it is essential to consider both age and axial length.

Although anti-VEGF therapy is currently recognized as the gold standard to treat nAMD [28,29], real-world data from a multicenter study in Japan suggest that approximately half of nAMD cases still require continuous anti-VEGF treatment, even after 2 years of treatment and an extended regimen [30]. Understanding the disease characteristics, particularly how choroidal thickness varies among AMD subtypes, may be beneficial in tailoring treatments to individual cases.

Despite several reports comparing the choroidal thickness between eyes with CSC and healthy eyes when using UWF SS-OCT, to the best of our knowledge, no study has investigated AMD using UWF SS-OCT. We aimed to evaluate the correlation between age and axial length in eyes with AMD, as well as to examine central and peripheral choroidal thickness through using UWF SS-OCT on the AMD subtypes.

## 2. Materials and Methods

### 2.1. Patients

This retrospective study was approved by the Institutional Review Board of Kyushu University, Fukuoka, Japan, and adhered to the tenets of the Declaration of Helsinki. All data were anonymized prior to analysis. We included patients with treatment-naïve AMD, including those with typical AMD (tAMD), PCV, PNV, and retinal angiomatous proliferation (RAP), who visited Kyushu University Hospital between March 2021 and March 2022. All patients underwent comprehensive ophthalmic examinations at the initial presentation for both eyes, including best-corrected visual acuity with a Landolt chart, slit-lamp biomicroscopy, intraocular pressure, color fundus photography covering the 45-degree posterior retina, fluorescein/indocyanine green angiography (FA/ICGA) (HRA-II; Heidelberg Engineering, Dossenheim, Germany), spectral-domain optical coherence tomography (SD-OCT) (Spectralis HRA+OCT or Cirrus 5000 HD-OCT; Zeiss, Dublin, CA, USA), UWF SS-OCT (Xephilio OCT-S1; Canon Medical Systems, Tokyo, Japan), and OCT angiography (OCTA) (AngioVue Imaging System, Optovue Inc., Fremont, CA, USA).

The nAMD subtype was diagnosed on the basis of fundoscopy, FA/ICGA, OCT, and OCTA at the initial visit. PCV was diagnosed based on the presence of the polypoidal dilatations revealed by ICGA and a sharp peak in the PED on the OCT images. The diagnosis of PNV was based on the absence of large drusen and the presence of either choroidal vascular hyperpermeability or underlying dilated choroidal vessels. PNV was defined as the presence of type 1 MNV as visualized by OCTA (Figure S1). Eyes with noisy UWF SS-OCT en face images were excluded (12 eyes of 12 patients, including 5 AMD, 3 PCV, 2 PNV, and 2 RAP patients).

### 2.2. Evaluations of Choroidal Thickness by UWF SS-OCT

The central and peripheral choroidal thicknesses were analyzed as described previously [21]. Briefly, we acquired three-dimensional volume data of a vertical 20 mm and horizontal 23 mm dimension, as well as a scan depth of 5.3 mm using UWF SS-OCT. For the segmentation of the choroid, we set the choroidal thickness as the vertical distance from Bruch’s membrane to the chorioscleral interface. Segmentation was automatically performed using built-in software (Xephilio OCT-S1). Our methods for choroidal thickness were similar to those in a previous study [21], except that we performed an automatic real-shape correction on the acquired OCT images through using software provided by Canon Medical Systems (US patent 9149181 B2) for more accurate evaluations of wide-field choroidal thicknesses [22].

To compare the central and peripheral choroidal thicknesses among the AMD subtypes, we set a grid consisting of three circles with diameters of 3 mm, 9 mm, and 18 mm, which were centered on the fovea. Three subfields were set in our study: (a) <3 mm, (b) <9 mm, and (c) 9–18 mm (Figure S2).

### 2.3. Statistical Analyses

Age, axial length, and choroidal thickness were analyzed using Dunnett’s test. Sex ratios were compared using Fisher’s exact test with a Bonferroni correction. Correlations between choroidal thickness and sex, age, and axial length were analyzed using Spearman’s correlation coefficients. The effects of age and axial length on choroidal thickness were analyzed using multivariate regression analysis, and the adjusted choroidal thicknesses among the AMD subtypes were compared via an analysis of covariance (ANCOVA).

## 3. Results

### 3.1. Characteristics and AMD Subtypes of the Enrolled Patients

One hundred and seventy-two eyes of 170 patients were enrolled. The mean ± standard deviation (SD) age of the patients was 72.8 ± 10.4 years, and there were 116 males and 54 females. We included 75 eyes with tAMD from patients with nAMD, 56 with PCV (54 patients), 29 with PNV, and 12 with RAP. There were no significant differences in the sex ratio and axial length among the AMD subtypes, whereas there was a significant difference in patient age between the PNV and tAMD groups (Table 1).

**Figure 1:**
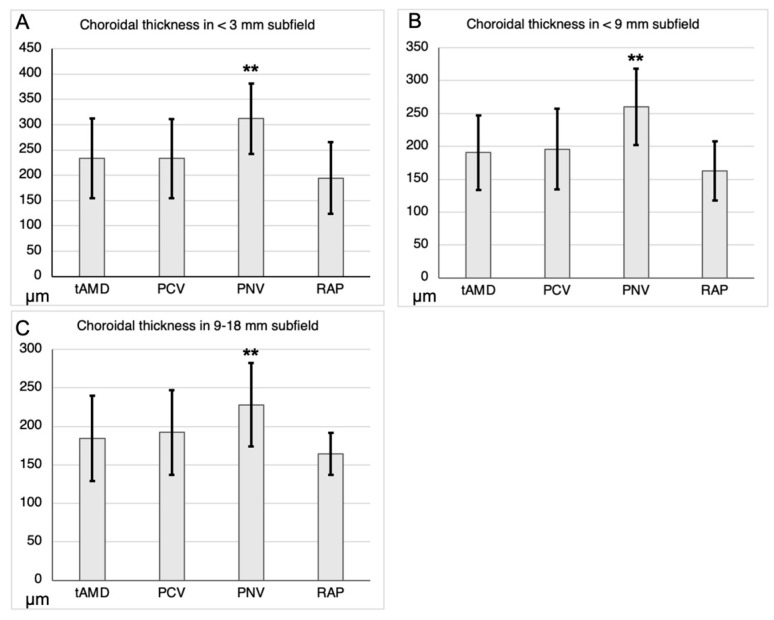
Choroidal thicknesses among the AMD subtypes in the central and peripheral subfields. The choroidal thickness of PNV was significantly greater than tAMD in all subfields. The choroidal thicknesses were analyzed by Dunnett’s test with tAMD as a reference (the mean ± SD, μm). (**A**) <3 mm subfield, (**B**) <9 mm subfield, and (**C**) 9–18 mm subfield. ** *p* < 0.01.

The mean ± SD choroidal thicknesses of tAMD, PCV, PNV, and RAP in the <3 mm subfield were 233.4 ± 78.5, 233.1 ± 78.0, 312.0 ± 69.3, and 194.6 ± 71.0 μm, respectively. The mean ± SD choroidal thicknesses of tAMD, PCV, PNV, and RAP in the <9 mm subfield were 190.6 ±56.9, 195.8 ± 61.3, 259.8 ± 58.1, and 162.9 ± 45.0 μm, respectively. The mean ± SD choroidal thicknesses of tAMD, PCV, PNV, and RAP in the 9–18 mm subfield were 184.3 ± 55.6, 192.2 ± 55.2, 228.1 ± 53.9, and 164.4 ± 27.6 μm, respectively (Figure 1). The choroidal thickness of the PNV group was significantly greater than that of the tAMD group in all subfields, whereas that of the PCV and RAP did not differ significantly from that of the tAMD group.

### 3.2. Choroidal Thickness in the Central, Posterior, and Peripheral Subfields

Next, we examined the correlation of choroidal thickness with sex, age, and axial length. Significant negative correlations were observed between choroidal thickness and age and axial length (Table 2). The correlation coefficients between choroidal thickness and age were −0.36, −0.42, and −0.47 in the <3 mm, <9 mm, and 9–18 mm subfields, respectively (*p* < 0.01). Correlation coefficients between choroidal thickness and axial length were −0.28, −0.26, and −0.18 in the <3 mm, <9 mm, and 9–18 mm subfields, respectively (*p* < 0.01, *p* < 0.01, and *p* = 0.03, respectively). There were no significant correlations between choroidal thickness and the sex ratio in any subfield (Table 2). Thus, age significantly correlated with choroidal thickness in this cohort.

### 3.3. Choroidal Thickness Adjusted by the Age and Axial Length among the AMD Subtypes

**Figure 2:**
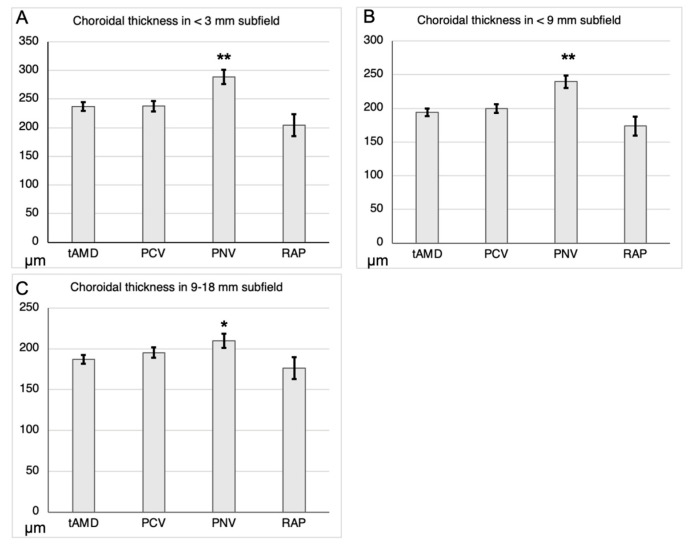
Comparison of choroidal thickness adjusted by age and axial length among AMD subtypes. Choroidal thickness adjusted by age and axial length in each subfield was compared with tAMD as reference by ANCOVA (the mean ± SE, μm). (**A**) <3 mm subfield, (**B**) <9 mm subfield, and (**C**) 9–18 mm subfield. * *p* < 0.05. ** *p* < 0.01.

Since choroidal thickness was significantly correlated with age and axial length, we compared them among the AMD subtypes. The mean ± standard error (SE) adjusted choroidal thicknesses of tAMD, PCV, PNV, and RAP in the <3 mm central subfield were 237.3 ± 7.6, 237.7 ± 8.8, 288.7 ± 12.6, and 204.7 ± 19.2 μm, respectively; of tAMD, PCV, PNV, and RAP in the <9 mm posterior subfield were 193.8 ± 5.6, 199.6 ± 6.5, 239.6 ± 9.3, and 173.9 ± 14.1 μm, respectively; and that of tAMD, PCV, PNV, and RAP in the 9–18 mm peripheral subfield were 187.0 ± 5.3, 195.4 ± 6.2, 210.0 ± 8.8, and 176.4 ± 13.4 μm, respectively. Thus, even after adjusting for age and axial length, the choroidal thickness of PNV—but not of PCV or RAP—was significantly greater than that of tAMD in the <3 mm (*p* < 0.01), <9 mm (*p* < 0.01), and 9–18 mm subfields (*p* = 0.03, ANCOVA) (Figure 2).

Since the differences in the central and posterior choroidal thicknesses between PNV and tAMD were statistically more significant than those in the periphery (Figure 2), we compared the ratios of the posterior (<9 mm) to peripheral (9–18 mm) choroidal thicknesses among the AMD subtypes. The mean ± SD ratio of the posterior <9 mm to peripheral 9–18 mm choroidal thicknesses of tAMD, PCV, PNV, and RAP were 1.04 ± 0.15, 1.02 ± 0.16, 1.15 ± 0.13, and 0.98 ± 0.16 μm, respectively (Table 3). Notably, the ratio of posterior choroidal thickness (<9 mm) to peripheral choroidal thickness (9–18 mm) in PNV was significantly greater than that in tAMD (*p* < 0.01, Dunnett’s test). This indicates that PNV may be associated with a thickened choroid, particularly in the posterior region.

## 4. Discussion

We examined the differences in the central and peripheral choroidal thicknesses among the AMD subtypes using UWF SS-OCT imaging. A previous study indicated that eyes with AMD exhibit hyperfluorescent deep choroidal veins in UWF ICGA when compared to unaffected or healthy eyes [31]. It has also been reported that PNV and PCV are characterized by thick choroid and intervortex venous anastomoses [32]. These findings suggest that the abnormal dilation of deep choroidal vessels, including those in the periphery, may play a role in the pathology of PNV or PCV. However, studies that have thoroughly analyzed both the posterior and peripheral choroidal thicknesses using UWF-OCT are limited to AMD. Regarding the interpretation of choroidal thickness data in AMD, it is important to note that choroidal thickness varies depending on several factors such as age and axial length [26]. Age is particularly important when considering the choroidal thickness among AMD subtypes. RAP was associated with thinner choroids [33]. However, RAP tends to occur in older individuals; therefore, it is possible that a thin choroid might simply be due to older age. Similarly, this could also be argued in cases of PNV, which tend to occur in younger individuals [34]. Therefore, to determine whether there were significant differences in the choroidal thicknesses among the disease types, we considered the effects of age and axial length when comparing the choroidal thicknesses among the different AMD subtypes. Consistently, our results demonstrated that both central and peripheral choroidal thicknesses were significantly correlated with age and axial length, and that there were significant differences in the central and peripheral choroidal thicknesses between PNV and tAMD even after adjusting for age and axial length. To our knowledge, this is the first study to examine the differences in the central/peripheral choroidal thickness among AMD subtypes.

Previous reports on CSC have shown that the entire choroid is thickened in CSC compared to healthy eyes, which is particularly evident in the posterior choroid [21,22,35,36]. In previous studies, there was no difference in age between the CSC group and healthy controls. However, we found a significant difference in age among the AMD subtypes, such as patients with PNV were younger. However, even when accounting for age and axial length, the choroidal thickness in the PNV group was still significantly greater than that in the tAMD group, whereas no such difference was noted in the PCV group. Furthermore, the thickened choroid in the PNV group was more pronounced in the posterior subfield (<9 mm) than in the peripheral subfield (9–18 mm) (Table 3). A similar finding was reported in that the choroid in the macular/posterior area was particularly thickened compared with the periphery in CSC [21,22,35,36]. Our diagnosis of PNV was based on the detection of MNV using OCTA, which clearly differentiated the eyes diagnosed with PNV from those with CSC. Overall, our results indicate that the choroidal thickness in PNV is greater than that in other AMD subtypes, particularly in the posterior pole rather than the periphery.

RAP occurs more frequently in older individuals and is associated with thin choroids [33]. In this study, the ratio of posterior (<9 mm) to peripheral choroidal thickness (9–18 mm) was relatively lower in RAP patients (Table 3), although no significant differences were observed (which is likely due to the small number of RAP patients). This suggests that, at disease onset, the choroid may already be thin across the entire area in patients with RAP. In healthy individuals, the posterior choroid is typically thicker than the peripheral choroid [22,37]. However, in this study, the ratio of posterior to peripheral choroidal thickness in RAP was less pronounced than in the other subtypes. This may be because the thickening of the choroid, associated with larger vessels near the vortex vein in the periphery [38,39], may have less of an impact on patients with RAP.

Several genome-wide association studies (GWAS) have been conducted to investigate the causal factors related to the onset of AMD/CSC. While AMD and CSC share several genetic risk factors such as *TNFRSF10A* [40,41], *CFH* risk alleles for AMD have been shown to be protective against CSC [40,42,43], indicating that at least a part of the genetic backgrounds in AMD and CSC may be different. Hosoda et al. reported an association of two susceptibility loci, rs800292 in *CFH* and rs3793217 in *VIPR2*, with choroidal thickness and CSC development [40]. Importantly, the relationship of the genetic backgrounds between AMD and PNV is not yet fully understood. While this study did not examine genetic risk factors, one might think that PNV and CSC might share some genetic backgrounds, as well as characteristics in choroidal thickness. Future GWAS studies on risk factors for PNV are essential.

This study has several limitations. This was a single-center, retrospective study with a small sample size. As the study only included Japanese participants, the generalizability of the findings to other ethnic groups remains unknown. Choroidal thickness follows circadian rhythms, being thickest at night due to increased blood flow from minimal light stimulation, and thinnest in the morning when light stimulation reduces blood flow. The maximum difference in the choroidal thickness was 3% [44]. We collected OCT data from the patients between 9 AM and noon to minimize the influence of these circadian variations.

Another limitation of our study was the insufficient angle of view to visualize the vortex vein ampulla on OCT imaging. Understanding the changes in the deep choroidal vasculature, including vortex veins, is crucial in the pathology of AMD subtypes, including PNV, particularly in the context of the venous overload choroidopathy hypothesis [12]. However, owing to the limited field of view of the UWF SS-OCT, which is obtained from the patients’ front view in this study, the exact evaluation of choroidal thickness, including the vortex vein ampulla, was not sufficient. Acknowledging this limitation highlights the importance of further investigations utilizing imaging modalities that provide a more expanded field of view. In previous studies using conventional non-wide-field OCT, it was reported that the peripapillary choroidal thickness is thicker in African or Hispanic populations compared to European populations [45,46]. Since this study focused exclusively on Japanese individuals, further studies on the relationship between race and choroidal thickness with UWF-OCT analysis will be required. Additionally, this study analyzed only treatment-naïve cases. Further investigations are needed to understand how these findings change with treatment interventions.

The significant age differences among AMD subtypes suggest that a specific type of AMD might develop with patient age over time. It has been hypothesized that CSC may evolve into PNV, which may then progress to PCV [4,47]. A retrospective analysis revealed that the development of type 1 MNV was observed in 15.6% of CSC cases. Similar to CSC, our findings revealed that, in PNV, the posterior choroid tends to be thicker than the peripheral choroid. This supports the hypothesis that CSC may evolve into PNV over time. Although some PNV patients do not have a history of CSC, it is plausible that a younger age of onset may lead to CSC, whereas an older age of onset could result in PNV, with both conditions potentially sharing similar pathologies. Hence, our analysis revealed the wide-field characteristics of the posterior and peripheral choroids in PNV, which may differ from those observed in PCV and tAMD.

## Figures and Tables

**Table 1:** Patient characteristics and AMD subtypes.

	tAMD	PCV	PNV	RAP	*p*-Value
*N*	75	54	29	12	
Female, *n* (%)	20 (26.7%)	18 (33.3%)	10 (34.5%)	7 (58.3%)	NS
Age (y)	73.4 ± 10.4	73.6 ± 8.9	67.1 ± 12.4	78.3 ± 6.7	1.00, 0.01, 0.30
Axial length (mm)	24.7 ± 1.8	24.7 ± 1.2	24.3 ± 1.1	24.3 ± 1.1	1.00, 0.60, 0.72

Age and axial length were analyzed using Dunnett’s test, with tAMD as a reference. The *p*-values for PCV, PNV, and RAP are shown. Sex ratios were analyzed using Fisher’s exact test with the Bonferroni correction.

**Table 2:** Correlations between choroidal thickness and sex/age/axial length.

	Correlation Coefficient			*p*-Value
	Subfield			
	<3 mm	<9 mm	9–18 mm	
Sex	−0.07	−0.06	0.02	0.50, 0.45, 0.88
Age	−0.36	−0.42	−0.47	<0.01, <0.01, <0.01
Axial length	−0.28	−0.26	−0.18	<0.01, <0.01, 0.03

Correlations between choroidal thickness and sex, age, and axial length were evaluated using the Spearman’s rank correlation coefficient. *p*-values are shown for the subfields of <3, <9, and 9–18 mm, respectively.

**Table 3:** Ratios of the posterior to peripheral choroidal thicknesses among the AMD subtypes.

	tAMD	PCV	PNV	RAP
Ratio of <9 mm to 9–18 mm choroidal thickness	1.04 ± 0.15	1.02 ± 0.16	1.15 ± 0.13	0.98 ± 0.16
*p*-value		0.82	<0.01	0.53

The ratios of posterior to peripheral choroidal thicknesses among the AMD subtypes were analyzed using Dunnett’s test, with tAMD as the reference (the mean ± SD). The ratio of posterior (<9 mm) to peripheral (9–18 mm) choroidal thicknesses in PNV was significantly greater than that in tAMD (*p* < 0.01).

## Data Availability

All data generated or analyzed during this study are included in this article. Further inquiries can be directed to the corresponding authors.

## Supplementary Materials

The following supporting information can be downloaded at: https://www.mdpi.com/article/10.3390/jcm12165364/s1, Figure S1: A representative OCTA image of PNV; Figure S2: Choroidal thickness map and subfields divided by circle grids.

## References

[1] Wong W.L., Su X., Li X., Cheung C.M., Klein R., Cheng C.Y., Wong T.Y. (2014). Global prevalence of age-related macular degeneration and disease burden projection for 2020 and 2040: A systematic review and meta-analysis. Lancet Glob. Health.

[2] Klein R., Klein B.E., Knudtson M.D., Wong T.Y., Cotch M.F., Liu K., Burke G., Saad M.F., Jacobs D.R. (2006). Prevalence of age-related macular degeneration in 4 racial/ethnic groups in the multi-ethnic study of atherosclerosis. Ophthalmology.

[3] Coscas G., Yamashiro K., Coscas F., De Benedetto U., Tsujikawa A., Miyake M., Gemmy Cheung C.M., Wong T.Y., Yoshimura N. (2014). Comparison of exudative age-related macular degeneration subtypes in Japanese and French Patients: Multicenter diagnosis with multimodal imaging. Am. J. Ophthalmol..

[4] Yanagi Y., Foo V.H.X., Yoshida A. (2019). Asian age-related macular degeneration: From basic science research perspective. Eye.

[5] Yanagi Y. (2020). Pachychoroid disease: A new perspective on exudative maculopathy. Jpn. J. Ophthalmol..

[6] Gallego-Pinazo R., Dolz-Marco R., Gómez-Ulla F., Mrejen S., Freund K.B. (2014). Pachychoroid diseases of the macula. Med. Hypothesis Discov. Innov. Ophthalmol..

[7] Takahashi A., Ooto S., Yamashiro K., Tamura H., Oishi A., Miyata M., Hata M., Yoshikawa M., Yoshimura N., Tsujikawa A. (2018). Pachychoroid Geographic Atrophy: Clinical and Genetic Characteristics. Ophthalmol. Retina.

[8] Pang C.E., Freund K.B. (2015). Pachychoroid neovasculopathy. Retina.

[9] Spaide R.F. (2018). Disease expression in nonexudative age-related macular degeneration varies with choroidal thickness. Retina.

[10] Hiroe T., Kishi S. (2018). Dilatation of Asymmetric Vortex Vein in Central Serous Chorioretinopathy. Ophthalmol. Retina.

[11] Shiihara H., Sonoda S., Terasaki H., Kakiuchi N., Yamashita T., Uchino E., Murao F., Sano H., Mitamura Y., Sakamoto T. (2020). Quantitative analyses of diameter and running pattern of choroidal vessels in central serous chorioretinopathy by en face images. Sci. Rep..

[12] Spaide R.F., Gemmy Cheung C.M., Matsumoto H., Kishi S., Boon C.J.F., van Dijk E.H.C., Mauget-Faysse M., Behar-Cohen F., Hartnett M.E., Sivaprasad S. (2022). Venous overload choroidopathy: A hypothetical framework for central serous chorioretinopathy and allied disorders. Prog. Retin. Eye Res..

[13] Cheung C.M.G., Lai T.Y.Y., Ruamviboonsuk P., Chen S.J., Chen Y., Freund K.B., Gomi F., Koh A.H., Lee W.K., Wong T.Y. (2018). Polypoidal Choroidal Vasculopathy: Definition, Pathogenesis, Diagnosis, and Management. Ophthalmology.

[14] Dansingani K.K., Gal-Or O., Sadda S.R., Yannuzzi L.A., Freund K.B. (2018). Understanding aneurysmal type 1 neovascularization (polypoidal choroidal vasculopathy): A lesson in the taxonomy of ‘expanded spectra’—A review. Clin. Exp. Ophthalmol..

[15] Uyama M., Wada M., Nagai Y., Matsubara T., Matsunaga H., Fukushima I., Takahashi K., Matsumura M. (2002). Polypoidal choroidal vasculopathy: Natural history. Am. J. Ophthalmol..

[16] Spaide R.F., Fisher Y.L., Ngo W.K., Barbazetto I. (2022). Regional scleral thickness as a risk factor for central serous chorioretinopathy. Retina.

[17] Kishi S., Matsumoto H. (2022). A new insight into pachychoroid diseases: Remodeling of choroidal vasculature. Graefes Arch. Clin. Exp. Ophthalmol..

[18] Zeng Q., Yao Y., Tu S., Zhao M. (2022). Quantitative analysis of choroidal vasculature in central serous chorioretinopathy using ultra-widefield swept-source optical coherence tomography angiography. Sci. Rep..

[19] Choma M., Sarunic M., Yang C., Izatt J. (2003). Sensitivity advantage of swept source and Fourier domain optical coherence tomography. Opt. Express.

[20] Spaide R.F., Koizumi H., Pozzoni M.C. (2008). Enhanced depth imaging spectral-domain optical coherence tomography. Am. J. Ophthalmol..

[21] Ishikura M., Muraoka Y., Nishigori N., Takahashi A., Miyake M., Ueda-Arakawa N., Miyata M., Ooto S., Tsujikawa A. (2022). Widefield Choroidal Thickness of Eyes with Central Serous Chorioretinopathy Examined by Swept-Source OCT. Ophthalmol. Retina.

[22] Funatsu R., Sonoda S., Terasaki H., Shiihara H., Mihara N., Horie J., Sakamoto T. (2023). Choroidal morphologic features in central serous chorioretinopathy using ultra-widefield optical coherence tomography. Graefes Arch. Clin. Exp. Ophthalmol..

[23] Imanaga N., Terao N., Nakamine S., Tamashiro T., Wakugawa S., Sawaguchi K., Koizumi H. (2021). Scleral Thickness in Central Serous Chorioretinopathy. Ophthalmol. Retina.

[24] Matsumoto H., Hoshino J., Mukai R., Nakamura K., Kishi S., Akiyama H. (2022). Clinical characteristics and pachychoroid incidence in Japanese patients with neovascular age-related macular degeneration. Sci. Rep..

[25] Shao L., Xu L., Chen C.X., Yang L.H., Du K.F., Wang S., Zhou J.Q., Wang Y.X., You Q.S., Jonas J.B. (2013). Reproducibility of subfoveal choroidal thickness measurements with enhanced depth imaging by spectral-domain optical coherence tomography. Investig. Ophthalmol. Vis. Sci..

[26] Ooto S., Hangai M., Yoshimura N. (2015). Effects of sex and age on the normal retinal and choroidal structures on optical coherence tomography. Curr. Eye Res..

[27] Xie J., Ye L., Chen Q., Shi Y., Hu G., Yin Y., Zou H., Zhu J., Fan Y., He J. (2022). Choroidal Thickness and Its Association With Age, Axial Length, and Refractive Error in Chinese Adults. Investig. Ophthalmol. Vis. Sci..

[28] Ying G.S., Huang J., Maguire M.G., Jaffe G.J., Grunwald J.E., Toth C., Daniel E., Klein M., Pieramici D., Wells J. (2013). Baseline predictors for one-year visual outcomes with ranibizumab or bevacizumab for neovascular age-related macular degeneration. Ophthalmology.

[29] Maguire M.G., Martin D.F., Ying G.S., Jaffe G.J., Daniel E., Grunwald J.E., Toth C.A., Ferris F.L., Fine S.L. (2016). Five-Year Outcomes with Anti-Vascular Endothelial Growth Factor Treatment of Neovascular Age-Related Macular Degeneration: The Comparison of Age-Related Macular Degeneration Treatments Trials. Ophthalmology.

[30] Ohji M., Takahashi K., Okada A.A., Kobayashi M., Matsuda Y., Terano Y. (2020). Efficacy and Safety of Intravitreal Aflibercept Treat-and-Extend Regimens in Exudative Age-Related Macular Degeneration: 52- and 96-Week Findings from ALTAIR: A Randomized Controlled Trial. Adv. Ther..

[31] Maruyama-Inoue M., Yamane S., Satoh H., Sato S., Kadonosono K. (2017). Choroidal angioarchitecture according to ultra-widefield indocyanine green angiography in age-related macular degeneration. J. VitreoRetin. Dis..

[32] Matsumoto H., Hoshino J., Mukai R., Nakamura K., Kikuchi Y., Kishi S., Akiyama H. (2020). Vortex Vein Anastomosis at the Watershed in Pachychoroid Spectrum Diseases. Ophthalmol. Retina.

[33] Yamazaki T., Koizumi H., Yamagishi T., Kinoshita S. (2014). Subfoveal choroidal thickness in retinal angiomatous proliferation. Retina.

[34] Kuranami A., Maruko R., Maruko I., Hasegawa T., Iida T. (2023). Pachychoroid neovasculopathy has clinical properties that differ from conventional neovascular age-related macular degeneration. Sci. Rep..

[35] Izumi T., Maruko I., Kawano T., Sakaihara M., Iida T. (2022). Morphological differences of choroid in central serous chorioretinopathy determined by ultra-widefield optical coherence tomography. Graefes Arch. Clin. Exp. Ophthalmol..

[36] Nishihara S., Maruko I., Izumi T., Kawano T., Iida T. (2022). Peripheral choroidal thickness determined by wide-field optical coherence tomography in eyes with central serous chorioretinopathy. Retina.

[37] Funatsu R., Sonoda S., Terasaki H., Shiihara H., Mihara N., Horie J., Sakamoto T. (2023). Normal peripheral choroidal thickness measured by widefield optical coherence tomography. Retina.

[38] Kakiuchi N., Terasaki H., Sonoda S., Shiihara H., Yamashita T., Tomita M., Shinohara Y., Sakoguchi T., Iwata K., Sakamoto T. (2019). Regional Differences of Choroidal Structure Determined by Wide-Field Optical Coherence Tomography. Investig. Ophthalmol. Vis. Sci..

[39] Tan C.S., Cheong K.X., Lim L.W., Li K.Z. (2014). Topographic variation of choroidal and retinal thicknesses at the macula in healthy adults. Br. J. Ophthalmol..

[40] Hosoda Y., Yoshikawa M., Miyake M., Tabara Y., Ahn J., Woo S.J., Honda S., Sakurada Y., Shiragami C., Nakanishi H. (2018). CFH and VIPR2 as susceptibility loci in choroidal thickness and pachychoroid disease central serous chorioretinopathy. Proc. Natl. Acad. Sci. USA.

[41] Akiyama M., Miyake M., Momozawa Y., Arakawa S., Maruyama-Inoue M., Endo M., Iwasaki Y., Ishigaki K., Matoba N., Okada Y. (2023). Genome-Wide Association Study of Age-Related Macular Degeneration Reveals 2 New Loci Implying Shared Genetic Components with Central Serous Chorioretinopathy. Ophthalmology.

[42] Miki A., Kondo N., Yanagisawa S., Bessho H., Honda S., Negi A. (2014). Common variants in the complement factor H gene confer genetic susceptibility to central serous chorioretinopathy. Ophthalmology.

[43] de Jong E.K., Breukink M.B., Schellevis R.L., Bakker B., Mohr J.K., Fauser S., Keunen J.E., Hoyng C.B., den Hollander A.I., Boon C.J. (2015). Chronic central serous chorioretinopathy is associated with genetic variants implicated in age-related macular degeneration. Ophthalmology.

[44] Kinoshita T., Mitamura Y., Shinomiya K., Egawa M., Iwata A., Fujihara A., Ogushi Y., Semba K., Akaiwa K., Uchino E. (2017). Diurnal variations in luminal and stromal areas of choroid in normal eyes. Br. J. Ophthalmol..

[45] Rhodes L.A., Huisingh C., Johnstone J., Fazio M.A., Smith B., Wang L., Clark M., Downs J.C., Owsley C., Girard M.J. (2015). Peripapillary choroidal thickness variation with age and race in normal eyes. Investig. Ophthalmol. Vis. Sci..

[46] Yang H., Luo H., Gardiner S.K., Hardin C., Sharpe G.P., Caprioli J., Demirel S., Girkin C.A., Liebmann J.M., Mardin C.Y. (2019). Factors Influencing Optical Coherence Tomography Peripapillary Choroidal Thickness: A Multicenter Study. Investig. Ophthalmol. Vis. Sci..

[47] Yamashiro K., Yanagi Y., Koizumi H., Matsumoto H., Cheung C.M.G., Gomi F., Iida T., Tsujikawa A. (2022). Relationship between Pachychoroid and Polypoidal Choroidal Vasculopathy. J. Clin. Med..

