# Supplementary material for: Differences in Central and Peripheral Choroidal Thickness among the Subtypes of Age-Related Macular Degeneration in an Asian Population
Source: J Clin Med. 2023 Aug 18;12(16):5364. doi: 10.3390/jcm12165364 (PMC10455582; doi:10.3390/jcm12165364)
Supplement: Supplementary file 1 [file jcm-12-05364-s001.zip › jcm-2567842-supplementary.pdf]

Supplementary figures

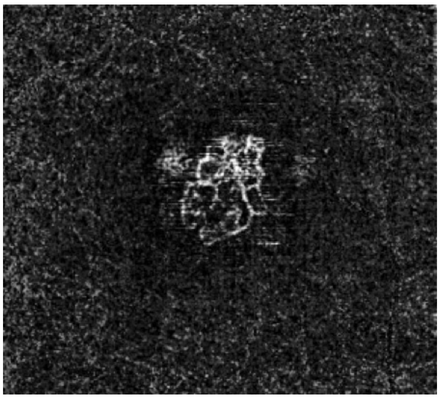

Figure S1. A representative OCTA image of PNV. MNV observed in a 3mm x 3mm OCTA image in a patient with PNV.

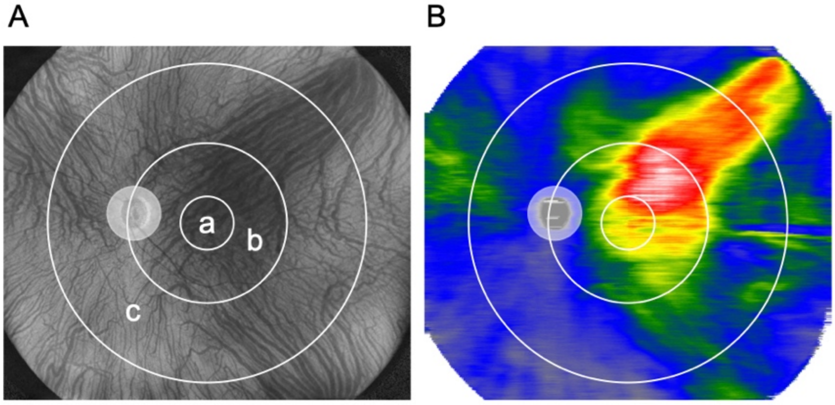

Figure S2. Choroidal thickness map and subfields divided by circle grids. Representative images of subfields and choroidal thickness map obtained by UWF SS-OCT. **A.** Three subfields are illustrated on an en face UWF SS-OCT image: < 3 mm subfield (a), < 9 mm subfield (b), and 9-18 mm subfield (c). **B.** A representative image of choroidal thickness map calculated by the Bruch's membrane to the chorioscleral interface in UWF OCT b-scans.
